# Supplementary material for: Clinical presentation of Oropouche virus infection: A systematic review and meta-analysis
Source: PLoS Negl Trop Dis. 2025 Apr 2;19(4):e0012962. doi: 10.1371/journal.pntd.0012962 (PMC11981143; doi:10.1371/journal.pntd.0012962)
Supplement: Attachment — Submitted filename: Response to Reviewers.docx [file pntd.0012962.s004.docx]

**Point-to-point responses to reviewers’ comments.**

We appreciate the referees’ assessment of our work, and their comments on aspects that could be improved. Below we respond to each referee’s points in detail, with notes as to where changes to the manuscript have been made.

Journal Requirements:

1) Please ensure that the CRediT author contributions listed for every co-author are completed accurately and in full.

At this stage, the following Authors/Authors require contributions: Yulei Li, Mengxi Gu, Xinyue Zhang, Xiaoying Zhu, Demin Cao, and Xidai Long. Please ensure that the full contributions of each author are acknowledged in the "Add/Edit/Remove Authors" section of our submission form.

The list of CRediT author contributions may be found here: https://journals.plos.org/plosntds/s/authorship#loc-author-contributions

Response: We sincerely appreciate your feedback. We have carefully reviewed and ensured that each coauthor's CRediT author contribution is accurate and complete.

2) Please provide an Author Summary. This should appear in your manuscript between the Abstract (if applicable) and the Introduction, and should be 150-200 words long. The aim should be to make your findings accessible to a wide audience that includes both scientists and non-scientists. Sample summaries can be found on our website under Submission Guidelines:

https://journals.plos.org/plosntds/s/submission-guidelines#loc-parts-of-a-submission

**Response:** Thanks for your suggestion. We have added Author Summary between the Abstract and the Introduction of our revised manuscript.

3) Please upload all main figures as separate Figure files in .tif or .eps format. For more information about how to convert and format your figure files please see our guidelines:

https://journals.plos.org/plosntds/s/figures

**Response:** Thanks for your suggestion. We have updated all main figures accordingly.

4) We have noticed that you have uploaded Supporting Information files, but you have not included a list of legends. Please add a full list of legends for your Supporting Information files after the references list.

**Response:** Thanks for your suggestion. We have added a full list of legends for our Supporting Information files accordingly.

5) We note that your Data Availability Statement is currently as follows: "The original contributions presented in the study are included in the article/supplementary material. Further inquiries can be directed to the corresponding author.". Please confirm at this time whether or not your submission contains all raw data required to replicate the results of your study. Authors must share the “minimal data set” for their submission. PLOS defines the minimal data set to consist of the data required to replicate all study findings reported in the article, as well as related metadata and methods (https://journals.plos.org/plosone/s/data-availability#loc-minimal-data-set-definition).

For example, authors should submit the following data:

- The values behind the means, standard deviations and other measures reported;

- The values used to build graphs;

- The points extracted from images for analysis..

Authors do not need to submit their entire data set if only a portion of the data was used in the reported study.

If your submission does not contain these data, please either upload them as Supporting Information files or deposit them to a stable, public repository and provide us with the relevant URLs, DOIs, or accession numbers. For a list of recommended repositories, please see https://journals.plos.org/plosone/s/recommended-repositories.

If there are ethical or legal restrictions on sharing a de-identified data set, please explain them in detail (e.g., data contain potentially sensitive information, data are owned by a third-party organization, etc.) and who has imposed them (e.g., an ethics committee). Please also provide contact information for a data access committee, ethics committee, or other institutional body to which data requests may be sent. If data are owned by a third party, please indicate how others may request data access.

**Response:** Thanks for your suggestion. We have submitted all the data accordingly.

Reviewers' comments:

Reviewer's Responses to Questions

Key Review Criteria Required for Acceptance?

As you describe the new analyses required for acceptance, please consider the following:

Methods

Reviewer #1: The objectives are clear and they have been achieved. Some comments:

"OROV is an acute febriel disease..." should be replaced by "OROV causes an acute febriel disease..." as OROV is the virus, nor the disease.

**Response:** Thanks for your suggestion. We have made the correction.

There is not and evident lack of literature; there is an evident lack of systematic review of the scattered data. So, better reformulate the sentence... "To date, the comprehensive.... as ther is an evident lack of systematic reviews."

**Response:** Thanks for your suggestion. We have made the correction.

Why does this lower limit of studies of less than five cases make these studies ineligible? They keep adding cases to the final "n".

**Response:** Thanks for your suggestion. Studies with fewer than five cases are excluded and widely used in numerous high - quality systematic reviews. Firstly, there is a lack of statistical power: when the sample size is extremely small, for example, fewer than five cases, it is extremely difficult to draw statistically significant conclusions. The results are more likely to be affected by random variations and outliers, and may not accurately represent the true relationships or phenomena under study. Secondly, a small sample size reduces the likelihood of generalizing the research findings to a larger population. Finally, too small a number of cases increases the risk of bias.

What meaning do the numbers 5 and 8 have next to Peru and Brazil, respectively, in Table 1? No information about this appears in the table key.

**Response:** Thanks for your suggestion. It was our inadvertent error and we have made a correction.

The phrase...."The prominence of ocular involvement, particularly retro-orbital pain, photophobia, and red eyes." lacks a clear meaning for me. It seems that the verb is missing.

**Response:** Thanks for your suggestion. These clinical symptom descriptors have been used in numerous reports in the literature, and we continue to use them here.

Remember that most taxonomic names are in italics. Check references 10, 16.

**Response:** Thanks for your suggestion. We have made the correction.

Reviewer #2: Are the objectives of the study clearly articulated with a clear testable hypothesis stated?

Yes, the objectives of the study are clearly articulated. The study aims to systematically review and meta-analyze the clinical presentation of Oropouche virus (OROV) infection. The hypothesis is implied rather than explicitly stated, but the study seeks to provide a comprehensive understanding of the clinical manifestations of OROV infection, which is a clear and testable objective.

**Response:** Thanks for your positive support.

Is the study design appropriate to address the stated objectives?

Yes, the study design is appropriate. The authors conducted a systematic review and meta-analysis, which is suitable for summarizing the clinical manifestations of OROV infection across multiple studies. The inclusion and exclusion criteria are well-defined, and the search strategy is comprehensive.

**Response:** Thanks for your positive support.

Is the population clearly described and appropriate for the hypothesis being tested?

Yes, the population is clearly described. The study includes 806 patients from 15 studies, all of whom were confirmed to have OROV infection via PCR or ELISA. The population is appropriate for the hypothesis being tested, as it focuses on individuals with confirmed OROV infection.

**Response:** Thanks for your positive support.

Is the sample size sufficient to ensure adequate power to address the hypothesis being tested?

Yes, the sample size of 806 patients from 15 studies is sufficient for a meta-analysis. The authors have included a reasonable number of studies and patients to ensure adequate power to address the hypothesis.

**Response:** Thanks for your positive support.

Were correct statistical analyses used to support conclusions?

Yes, the statistical analyses appear to be appropriate. The authors used a fixed-effect model for meta-analysis and assessed heterogeneity using the I² statistic. They also used forest plots and funnel plots to visualize the data and assess publication bias. The methods are well-described and appropriate for the type of data being analyzed.

**Response:** Thanks for your positive support.

Are there concerns about ethical or regulatory requirements being met?

No, there are no apparent concerns about ethical or regulatory requirements. The study is a systematic review and meta-analysis of previously published studies, so it does not involve direct human or animal subjects. The authors have declared no competing interests and no specific funding for this work.

**Response:** Thanks for your positive support.

Results

-Does the analysis presented match the analysis plan?

-Are the results clearly and completely presented?

-Are the figures (Tables, Images) of sufficient quality for clarity?

Reviewer #1: Appropriate and sufficient. If the quality and disaggregation of the clinical data in the different studies allowed it, a review of the clinical presentations by age or sex would have been interesting to read.

**Response:** Thanks for your positive support.

Reviewer #2: Does the analysis presented match the analysis plan?

Yes, the analysis presented matches the analysis plan outlined in the methods section. The authors conducted a meta-analysis of clinical symptoms and presented the results in a clear and structured manner.

**Response:** Thanks for your positive support.

Are the results clearly and completely presented?

Yes, the results are clearly and completely presented. The authors provide detailed tables and figures summarizing the prevalence of various symptoms, including general, gastrointestinal, ocular, respiratory, and skin-related symptoms. The results are well-organized and easy to follow.

**Response:** Thanks for your positive support.

Are the figures (Tables, Images) of sufficient quality for clarity?

Yes, the figures and tables are of sufficient quality for clarity. The forest plots and tables are well-constructed and provide a clear visual representation of the data. The figures are appropriately labeled and easy to interpret.

**Response:** Thanks for your positive support.

Conclusions

-Are the conclusions supported by the data presented?

-Are the limitations of analysis clearly described?

-Do the authors discuss how these data can be helpful to advance our understanding of the topic under study?

-Is public health relevance addressed?

Reviewer #1: The conclusion are supported by the data presented. Limitations of the analysis are clearly described but not completely assessed. Taking into account that the circulation of this virus basically occurs in Latin America, how many studies in Spanish or Portuguese/Brazilian have been not considered and therefore discarded? How many cases? What impact does this selection have on the final result?

**Response:** Thanks for your suggestion. We may have missed some studies in Spanish or Portuguese/Brazilian due to language limitations, but more high-quality studies are published in English , so we believe the conclusions of this study are solid.

The discussion is sufficient and obviously it has public health relevance for the professionals in the field.

**Response:** Thanks for your positive support.

Reviewer #2: Are the conclusions supported by the data presented?

Yes, the conclusions are supported by the data presented. The authors conclude that OROV infection presents with a wide range of symptoms, including fever, headache, gastrointestinal disturbances, ocular symptoms, and respiratory symptoms. These conclusions are well-supported by the meta-analysis results.

**Response:** Thanks for your positive support.

Are the limitations of analysis clearly described?

Yes, the limitations of the analysis are clearly described. The authors acknowledge that most of the included studies were retrospective and that there may be publication bias due to the exclusion of asymptomatic or mild cases. They also note the potential for selection bias and the lack of high-quality data in some studies.

**Response:** Thanks for your positive support.

Do the authors discuss how these data can be helpful to advance our understanding of the topic under study?

Yes, the authors discuss the public health relevance of their findings. They emphasize the need for heightened clinical awareness and a comprehensive approach to patient evaluation, which can help in the accurate diagnosis and management of OROV infection. This discussion is relevant and adds value to the study.

**Response:** Thanks for your positive support.

Is public health relevance addressed?

Yes, the public health relevance is addressed. The authors highlight the increasing incidence and geographic spread of OROV infections and the need for targeted public health interventions. They also discuss the importance of integrating ophthalmologic evaluations into the management protocol for suspected OROV cases.

**Response:** Thanks for your positive support.

Editorial and Data Presentation Modifications?

Use this section for editorial suggestions as well as relatively minor modifications of existing data that would enhance clarity. If the only modifications needed are minor and/or editorial, you may wish to recommend “Minor Revision” or “Accept”.

**Response:** Thanks for your positive support.

Reviewer #1: This reviewer believes that a new reading of the text and improving the constructions of some sentences would improve the manuscript.

**Response:** Thanks for your suggestion.

Reviewer #2: The authors should consider explicitly stating the hypothesis in the introduction to make the study's objectives even clearer.

**Response:** Thanks for your suggestion.

The discussion could be strengthened by comparing the findings with other arboviral infections (e.g., dengue, Zika) in more detail, as this would provide additional context for the clinical significance of OROV symptoms.

The authors should ensure that all abbreviations are defined upon first use (e.g., "OROV" is defined, but other abbreviations like "PCR" and "ELISA" are not).

**Response:** Thanks for your suggestion. We have made the correction accordingly.

Summary and General Comments

Use this section to provide overall comments, discuss strengths/weaknesses of the study, novelty, significance, general execution and scholarship. You may also include additional comments for the author, including concerns about dual publication, research ethics, or publication ethics. If requesting major revision, please articulate the new experiments that are needed.

Reviewer #1: This is a systematic review of the clinical symptoms of Oropouche virus infection based on previous work. Systematizes dispersed data and organizes it. Useful for case management and public health.

**Response:** Thanks for your positive support.

Reviewer #2: Strengths:

The study is well-designed and addresses an important gap in the literature regarding the clinical presentation of OROV infection.

**Response:** Thanks for your positive support.

The meta-analysis is conducted rigorously, with appropriate statistical methods and clear presentation of results.

**Response:** Thanks for your positive support.

The study has significant public health relevance, particularly in regions where OROV is endemic or emerging.

**Response:** Thanks for your positive support.

Weaknesses:

The study is limited by the retrospective nature of most included studies and potential publication bias, as acknowledged by the authors.

**Response:** Thanks for your suggestion.

The discussion could be expanded to provide more context on how OROV symptoms compare to other arboviral infections, which would enhance the study's clinical relevance.

**Response:** Thanks for your suggestion.

Novelty and Significance:

This is the first systematic review and meta-analysis to comprehensively summarize the clinical manifestations of OROV infection. The findings are novel and provide a foundational understanding that can guide future research and public health interventions.

**Response:** Thanks for your positive support.

General Execution and Scholarship:

The study is well-executed and demonstrates a high level of scholarship. The authors have followed appropriate guidelines for systematic reviews and meta-analyses, and the manuscript is well-written and organized.

**Response:** Thanks for your positive support.

PLOS authors have the option to publish the peer review history of their article (what does this mean?). If published, this will include your full peer review and any attached files.

If you choose “no”, your identity will remain anonymous but your review may still be made public.

Do you want your identity to be public for this peer review? For information about this choice, including consent withdrawal, please see our Privacy Policy.

Reviewer #1: No

Reviewer #2: No

protocols
